# Use of a high-speed pencil-tip burr for removal of well-fixed humeral components in revision shoulder arthroplasty

**DOI:** 10.1016/j.jsea.2026.100089

**Published:** 2026-09-11

**Authors:** John Abdelshaheed, Ryan Morrall, Zachary A. Grand, Albert D. Mousad, Rishi Chatterji, Casey M. Beleckas, Jonathan C. Levy

**Affiliations:** a Levy Shoulder to Hand Center at the Paley Orthopedic and Spine Institute, Boca Raton, FL, USA; b University of Central Florida College of Medicine, Orlando, FL, USA; c Florida Atlantic University Schmidt College of Medicine, Boca Raton, FL, USA; d Florida International University Herbert Wertheim College of Medicine, Miami, FL, USA; e Geisinger Musculoskeletal Institute, Wilkes-Barre, PA, USA

**Keywords:** Revision shoulder arthroplasty, Humeral component removal, High-speed burr, Humeral fracture, Outcomes, Total shoulder arthroplasty

## Abstract

**Background:**

Removal of a well-fixed humeral stemmed component during revision total shoulder arthroplasty (TSA) remains technically challenging and carries a risk of intra-operative fracture and bone loss. Various osteotomy-based methods have been described to facilitate extraction, though each carries its own morbidity. The high-speed pencil-tip burr offers an alternative method for controlled disruption of the bone–implant or cement–bone interface, potentially reducing complications and bone loss. The purpose of this study was to evaluate radiographic and clinical outcomes following revision TSA utilizing a high-speed pencil-tip burr for removal of well-fixed humeral components.

**Methods:**

A retrospective review of revision TSAs performed by a single surgeon between 2007 and 2023 was conducted. Clinical and radiographic data were collected via retrospective chart review in 2025. Patients were included if a high-speed pencil-tip burr was used for removal of a well-fixed humeral stem in the setting of revision TSA. Demographics, revision indications, adjunct instruments, and complications were collected from operative reports and institutional registry data. Clinical outcomes included the American Shoulder and Elbow Surgeons score, Simple Shoulder Test, Single Assessment Numeric Evaluation, and visual analog scale (VAS) scores for pain and function. Cases in which an intra-operative fracture occurred were reviewed for radiographic healing and functional recovery.

**Results:**

A total of 128 revision TSAs met inclusion criteria. The most common indications for revision were rotator cuff tears (56.3%) and glenoid loosening (36.7%), with multiple indications present in some patients. At a mean clinical follow-up of 43.4 ± 39.2 months, all patient-reported outcome measures improved significantly. Twenty-seven patients (21.1%) sustained intra-operative humeral fractures, including greater tuberosity (n = 15), surgical neck (n = 6), lesser tuberosity (n = 4), and humeral shaft (n = 2) fractures. Radiographically, 21 of 27 fractures (77.8%) demonstrated healing, with all non–greater tuberosity fractures healing and all cases of displacement (n = 4) or resorption (n = 2) occurring in greater tuberosity fractures. When compared with patients without fracture, those sustaining an intra-operative humeral fracture demonstrated no significant differences in post-operative American Shoulder and Elbow Surgeons, Simple Shoulder Test, Single Assessment Numeric Evaluation, VAS pain, VAS function, or range of motion.

**Conclusion:**

Use of a high-speed pencil-tip burr for removal of well-fixed humeral components during revision TSA allows reliable stem extraction without osteotomy, with an intra-operative fracture rate comparable to other series, favorable radiographic healing, and significant improvement in clinical and patient-reported outcomes.

There has been an exponential increase in both anatomic total shoulder arthroplasty (aTSA) and reverse total shoulder arthroplasty (rTSA) over the past 2 decades, primarily due to expanding indications and the growing elderly population. There is projected to be a 333% increase in shoulder arthroplasty demand among younger patients and more than a 700% increase among older patients by 2030.^9^ As the volume of shoulder arthroplasty continues to expand, the burden of revision surgery has risen in parallel. Studies depict an increase of 153% from 2012 to 2018, with linear regression predicting a continued rise in revision cases.^3^ This expanding revision burden makes the need for effective revision techniques increasingly important.

Removal of a well-fixed humeral stemmed component during revision surgeries is a difficult challenge and comes with an increased risk of complications such as intra-operative fracture, iatrogenic bone loss, and non-union of fractures and osteotomies. In order to mitigate these complications, surgical approaches, including the use of humeral windows, vertical humeral osteotomy, episiotomy, and longitudinal split osteotomy, have been developed in attempts to lower the rate of risks.^5^^,^^7^^,^^14^ Previous studies have reported favorable outcomes with these techniques for extraction of well-fixed humeral components. A high-speed pencil-tip burr offers an alternative adjunct to disrupt the cement–bone or implant–bone interface. This potentially allows for controlled extraction while minimizing the need for osteotomy. Despite its increasing use, outcomes associated with burr-assisted removal of well-fixed humeral stems remain under-reported.

The purpose of this study was to evaluate radiographic and clinical outcomes following revision total shoulder arthroplasty (TSA) in which a high-speed pencil-tip burr (Midas Rex 9TA30, Medtronic, Fort Worth, TX, USA) was used to aid removal of a well-fixed humeral component. Secondarily, outcomes and complications were analyzed among patients who sustained intra-operative humeral fractures, with emphasis on radiographic healing and functional recovery. We hypothesized that in patients undergoing revision shoulder arthroplasty for a well-fixed humeral stem, use of a high-speed pencil-tip burr would allow reliable extraction of the stem without need for osteotomy with acceptable rates of intra-operative fracture and post-operative clinical outcomes.

## Materials and methods

This is a retrospective cohort study of patients who underwent revision shoulder arthroplasty between 2007 and 2023 by a single fellowship-trained shoulder and elbow surgeon, in which a high-speed pencil-tip burr was used to remove a well-fixed humeral component.

Patients were included if they underwent revision TSA surgery requiring removal of a well-fixed humeral component using a high-speed pencil-tip burr. Patients with radiolucent lines around humeral stems on pre-operative radiographs and any patient with operative report description of a loose humeral component were excluded. This study was approved by an institutional review board (IRB #2025-061).

Demographic data including age, sex, and body mass index were recorded. Indications for revision, intra-operative adjunct instruments used, and post-operative complications were recorded from operative reports and the institutional shoulder and elbow registry. The subset of patients who sustained intra-operative humeral fractures was identified and radiographically reviewed to assess fracture location and evidence of healing at final follow-up. To minimize measurement bias, radiographic healing was independently assessed by 2 authors, with no disagreements. All procedures were performed by a single fellowship-trained surgeon using a consistent surgical technique to minimize technique-related variability.

### Surgical technique

All procedures were performed by a single fellowship-trained shoulder and elbow (J.C.L.). All revisions were performed through a standard deltopectoral approach with a subscapularis peel if the tendon was intact. In the setting of a well-fixed humeral stem, a high-speed pencil-tip burr (Midas Rex 9TA30, Medtronic, Fort Worth, TX, USA) was used along the bone–prosthetic or cement–prosthetic interface to facilitate controlled de-bonding of the stem (Fig. 1). This specific burr is low profile and side-cutting, allowing removal of minimal amount of bone or cement from the humeral prosthetic interface. Once the interface was adequately de-bonded, 1 of 2 extraction techniques were utilized for final stem removal: implant-specific broach handles or carbide tip punches. In cases where residual cement mantle remained following stem removal, osteotomes and cannulated taps were utilized. Long handle osteotomes helped to fracture sections of the cement mantle for intra-medullary removal. Alternatively, using the technique described by Berglund et al,^2^ sections of the cement mantle were sequentially removed by advancing a guide-wire and tap through the cement mantle. Management of intra-operative fractures was dependent on fracture pattern. Non-displaced humeral shaft fractures were managed with cerclage suture fixation, while greater tuberosity fractures were secured to the stem using sutures passed through the suture holes located on the rim and fins of the stem. Throughout this consecutive series of revision surgeries, no humeral osteotomy or cortical window was performed. Reconstruction was completed using an aTSA or rTSA shoulder system (Enovis/DJO, Austin, TX, USA) based upon the condition of the rotator cuff, with cemented or press-fit fixation chosen intra-operatively, at the surgeon's discretion based on observed bone quality and remaining bone support. The humeral stems removed in this cohort represented a variety of implant manufacturers and designs accumulated over the study period; the extraction technique described is not specific to any single implant system.

**Figure 1:**
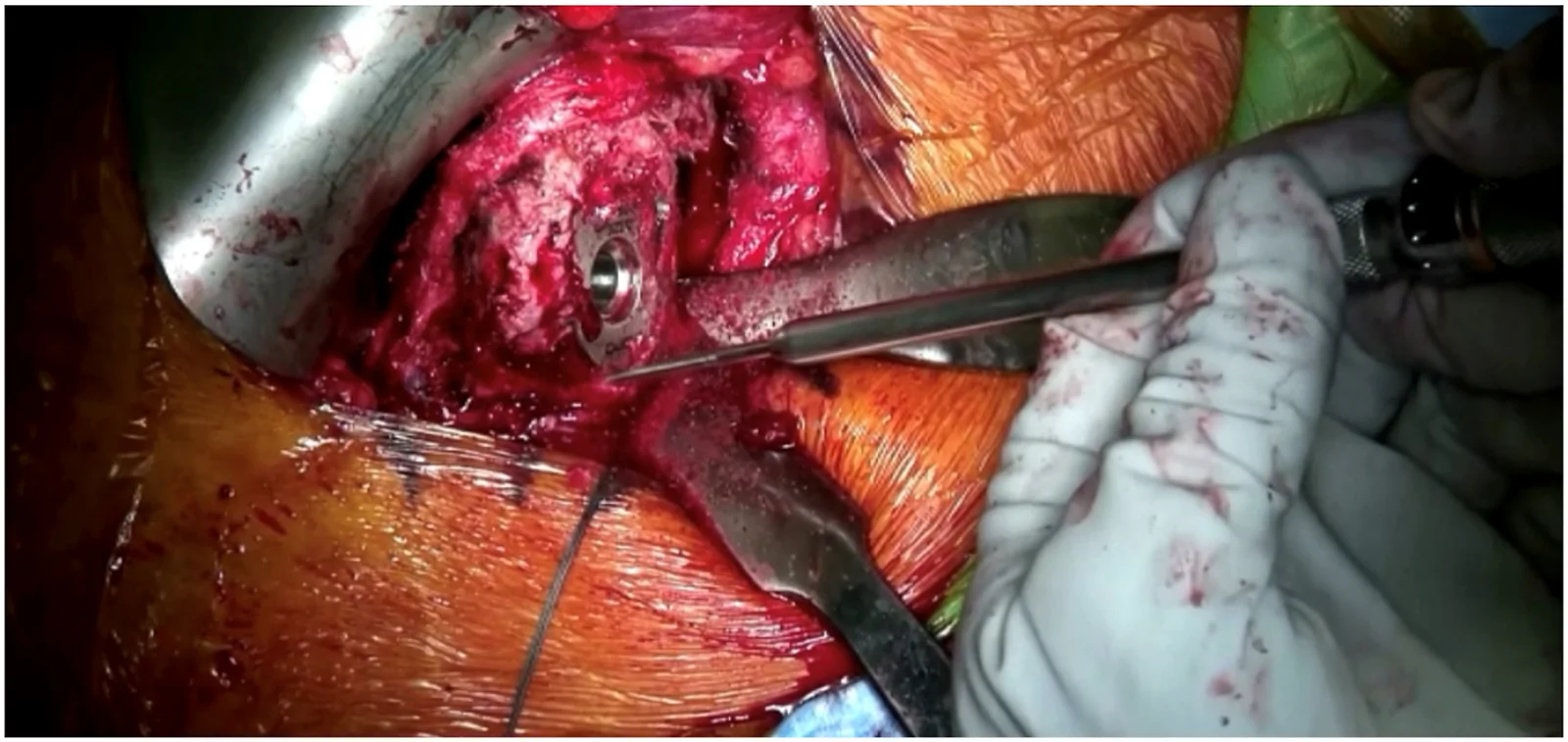
Intra-operative photograph demonstrating use of the high-speed pencil-tip burr to engage and remove the humeral component.

### Clinical outcomes

Patients were evaluated pre-operatively and at the most recent follow-up using the American Shoulder and Elbow Surgeons (ASES) score, Simple Shoulder Test (SST), Single Assessment Numeric Evaluation (SANE), visual analog scale (VAS) pain, and VAS function scores. Range of motion was measured by a physician or physician assistant using a goniometer for elevation and external rotation, and internal rotation was scored on a 10-point ordinal scale (buttock/greater trochanter = 2; sacrum–L4 = 4; L1–L3 = 6; T8–T12 = 8; T1–T7 = 10).^13^

### Radiographic analysis

Patients who sustained intra-operative humeral fractures had immediate post-operative radiographs (anteroposterior, Grashey, scapular Y, and axillary lateral) evaluated for fracture location and pattern. Most recent follow-up radiographs were analyzed for fracture healing and displacement. Healing was evaluated by 2 independent authors for agreement (C.M.B., J.C.L.); No disagreements occurred.

### Statistical analysis

Continuous variables were reported as mean ± standard deviation or median with interquartile range, and categorical variables as counts with percentages. Pre-operative and post-operative outcome measures were compared using paired *t*-tests or Wilcoxon signed-rank tests as appropriate after normality testing. To account for multiple comparisons across outcome measures, a Bonferroni correction was applied, yielding an adjusted significance threshold of *P* ≤ .002. Data were analyzed using IBM SPSS Statistics (version 29; IBM Corp., Armonk, NY, USA). Alpha was set at 0.05.

## Results

A total of 128 revision TSAs in which a high-speed pencil-tip burr was used for removal of a well-fixed humeral component were identified and included in the analysis (Fig. 2). During this same collection period, no patient required a humeral episiotomy or cortical window for humeral stem removal. The mean patient age was 70.5 ± 9.4 years, and 55% were male. The mean body mass index was 28.2 ± 6.4 kg/m^2^ (Table I).

**Figure 2:**
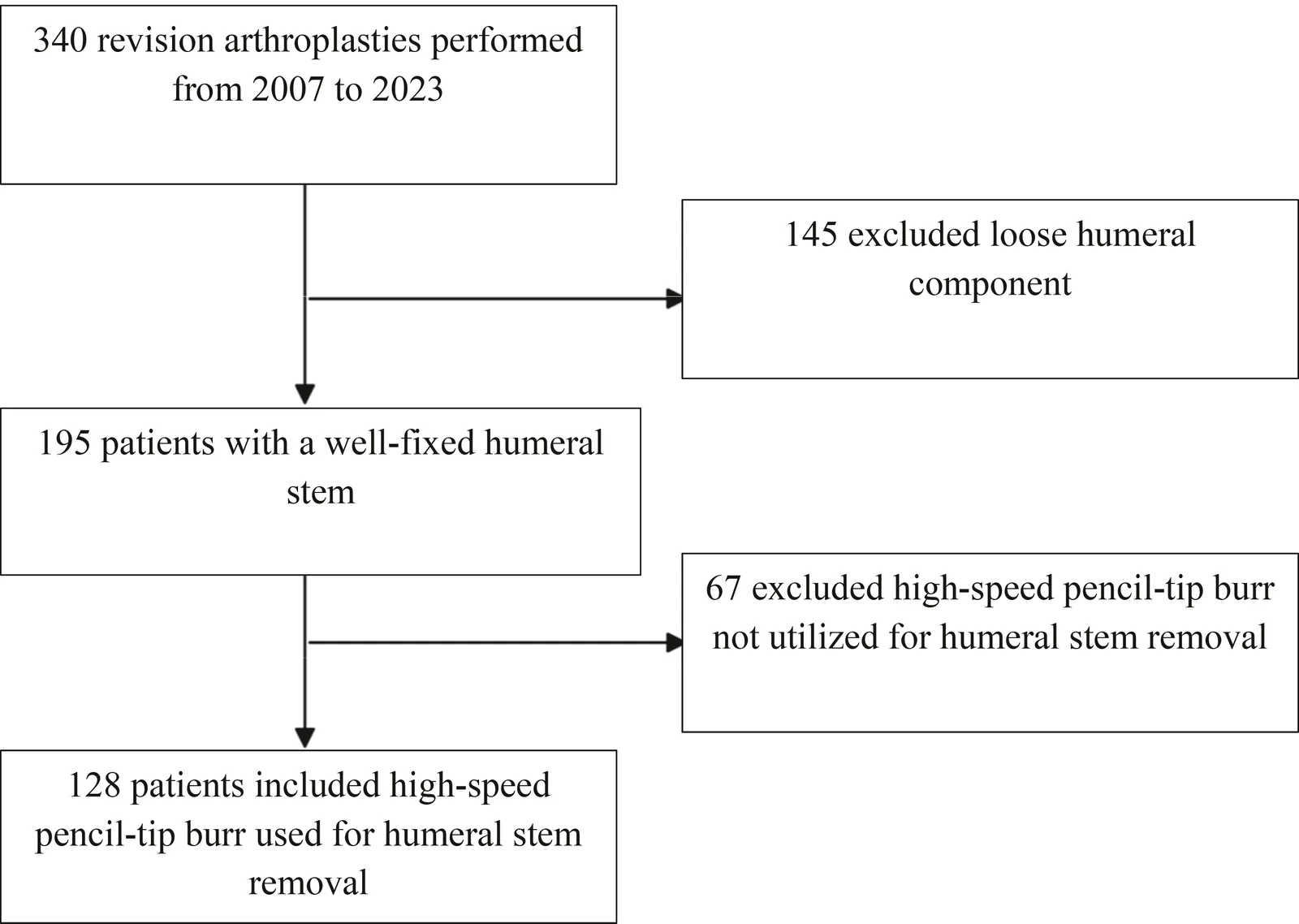
Strengthening the Reporting of Observational Studies in Epidemiology (STROBE) participant flow diagram.

**Table I:** Patient demographics and indications for revision shoulder arthroplasty

Variable	All patients (N = 128) Mean ± SD or n (%)
Age	70.5 ± 9.44
BMI (kg/m^2^)	28.2 ± 6.37
Sex	
Male	70 (54.7%)
Female	58 (45.3%)
Follow-up time (months)	43.40 ± 39.16
Indications for revision	
Cuff tear	72 (56.3%)
Glenoid loosening	47 (36.7%)
Infection	29 (22.7%)
Glenoid erosion	11 (8.6%)
Instability/dislocation	8 (6.3%)
Periprosthetic fracture	5 (3.9%)
Prior implant and revision type	
aTSA to rTSA	78 (60.9%)
Hemi-arthroplasty to rTSA	25 (19.5%)
rTSA to rTSA	5 (3.9%)
aTSA to antibiotic spacer (staged)	12 (9.4%)
Hemi-arthroplasty to antibiotic spacer (staged)	4 (3.1%)
rTSA to antibiotic spacer (staged)	4 (3.1%)

*aTSA*, anatomic total shoulder arthroplasty; *rTSA*, reverse total shoulder arthroplasty; *BMI*, body mass index; *SD*, standard deviation; *BMI*, body mass index.

Indications for revision were not mutually exclusive; percentages may exceed 100%.

The most common indications for revision (Table I) were rotator cuff tear (56.3%), glenoid loosening (36.7%), and infection (22.7%). Glenoid erosion (8.6%), instability/dislocation (6.3%), and periprosthetic fracture (3.9%) were less frequent. Multiple indications were present in some patients; therefore, percentages exceed 100%.

Prior implant and revision type (Table I) were as follows: 78 patients (60.9%) underwent revision from aTSA to rTSA, 25 (19.5%) from hemi-arthroplasty to rTSA, 5 (3.9%) from rTSA to rTSA, and 20 patients (15.6%) underwent staged revision with placement of an antibiotic spacer (12 from aTSA, 4 from hemi-arthroplasty, and 4 from rTSA).

At a mean clinical follow-up of 43.4 ± 39.2 months following revision, patients demonstrated significant improvement in all patient-reported outcome measures (*P* < .001; Table II). Forward elevation (*P* < .001) and internal rotation (*P* < .001) improved post-operatively, while external rotation remained unchanged (*P* = .222).

**Table II:** Pre-operative and post-operative patient-reported outcomes and range of motion

Variable	n	Pre-operative	Post-operative	*P* value
ASES	97	31.9 ± 19.8	58.9 ± 26.4	**<.001**
SST	98	2.42 ± 2.25	5.91 ± 3.48	**<.001**
SANE	98	27.8 ± 22.4	60.0 ± 30.7	**<.001**
VAS function	99	2.87 ± 2.09	6.25 ± 2.81	**<.001**
VAS pain	97	7.0 [4.0-8.0]	2.0 [0-6.0]	**<.001**
Range of motion				
Elevation	99	65° [45-95]	120° [80-139]	**<.001**
External rotation	98	30° [20-45]	30° [20-50]	.222
Internal rotation∗	84	4 [2-4]	4 [2-8]	**<.001**

*SANE*, Single Assessment Numeric Evaluation; IQR, interquartile range; *ASES*, American Shoulder and Elbow Surgeons; *SST*, Simple Shoulder Test; *VAS*, visual analog scale.

Values are reported as mean ± standard deviation unless otherwise indicated; median [interquartile range] is shown for non-normally distributed variables (VAS pain and range of motion). Pre-operative and Post-operative comparisons were performed using paired *t*-tests or Wilcoxon signed-rank tests, as appropriate. Bold values indicate statistical significance.

∗ Internal rotation was scored on a 10-point scale using the following conversions: buttock/greater trochanter = 2, sacrum–L4 = 4, L1–L3 = 6, T8–T12 = 8, T1–T7 = 10.

A total of 27 patients (21.1%) experienced an intra-operative humeral fracture (Table III), including greater tuberosity (n = 15; n = 7 displaced, n = 8 non-displaced), surgical neck (n = 6; n = 3 displaced, n = 3 non-displaced), lesser tuberosity (n = 4; n = 1 displaced, n = 3 non-displaced), and humeral shaft (n = 2; n = 0 displaced, n = 2 non-displaced) fractures. The majority involved the greater tuberosity. Of the cases where a humeral fracture occurred, 21 of 27 fractures (77.8%) demonstrated radiographic healing. All non–greater tuberosity fractures healed. Of the 6 greater tuberosity fractures that did not heal, 4 demonstrated displacement and 2 underwent resorption over time.

**Table III:** Intra-operative humeral fracture characteristics and radiographic outcomes (n = 27)

Variable	n (%)
Fracture location	
Greater tuberosity	15 (55.6%)
Displaced	7
Non-displaced	8
Lesser tuberosity	6 (22.2%)
Displaced	3
Non-displaced	3
Proximal humerus	4 (14.8%)
Displaced	1
Non-displaced	3
Humeral shaft	2 (7.4%)
Displaced	0
Non-displaced	2
Radiographic outcome	
Healed	21 (77.8%)
Displaced	4 (14.8%)
Resorbed	2 (7.4%)

Among the 27 patients who sustained an intra-operative humeral fracture, significant improvement in all patient-reported outcome measures was observed at latest follow-up (Table IV). ASES (*P* < .001), SST (*P* = .002), SANE (*P* < .001), VAS pain (*P* < .001), and VAS function (*P* < .001) scores all improved significantly post-operatively. Forward elevation (*P* = .003), external rotation (*P* = .030) and internal rotation (*P* = .200) remained unchanged.

**Table IV:** Pre-operative and post-operative patient-reported outcomes and range of motion in patients with intra-operative humeral fracture

Variable	Pre-operative	Post-operative	*P* value
ASES	24.6 ± 14.1	59.0 ± 24.2	**<.001**
SST	2.0 [1.0-3.3]	6.0 [2.5-9.5]	**.002**
SANE	26.6 ± 16.4	69.2 ± 26.7	**<.001**
VAS pain	8.0 [5.0-9.0]	3.5 [0.0-6.3]	**<.001**
VAS function	3.0 [1.8-4.0]	7.5 [5.0-9.0]	**<.001**
Range of motion			
Elevation	55 [44-80]	120 [80-135]	.003
External rotation	30 [19-41]	25 [0-30]	.030
Internal rotation∗	4 [2.0-8]	4 [2-8]	.200

*SANE*, Single Assessment Numeric Evaluation; IQR, interquartile range; *ASES*, American Shoulder and Elbow Surgeons; *SST*, Simple Shoulder Test; *VAS*, visual analog scale.

Values are reported as mean ± standard deviation unless otherwise indicated; median [interquartile range] is shown for non-normally distributed variables (VAS pain and range of motion). Pre-operative and Post-operative comparisons were performed using paired *t*-tests or Wilcoxon signed-rank tests, as appropriate.

∗ Internal rotation was scored on a 10-point scale using the following conversions: buttock/greater trochanter = 2, sacrum–L4 = 4, L1–L3 = 6, T8–T12 = 8, T1–T7 = 10.

When post-operative outcomes were compared between patients with and without intra-operative humeral fracture (Table V), no significant differences were observed in ASES, SST, SANE, VAS pain, or VAS function scores. Forward elevation, external rotation, and internal rotation were also similar between cohorts.

**Table V:** Comparison of post-operative outcomes in patients with and without intra-operative humeral fracture

Variable	No fracture	Fracture	*P* value
ASES	61.2 ± 26.9	61.1 ± 26.4	.986
SST	6.0 [3.5-9.0]	6.0 [3.0-8.0]	.910
SANE	61.9 ± 31.0	67.8 ± 28.0	.438
VAS pain	3.0 [0.0-5.5]	4.5 [0.0-7.0]	.636
VAS function	7.0 [4.0-8.75]	7.5 [5.0-9.0]	.410
Range of motion			
Elevation	123 [90-145]	120 [60-135]	.465
External rotation	40 [30-50]	23 [0-30]	.004
Internal rotation∗	4 [2-8]	4.0 [2-8]	.238

*SANE*, Single Assessment Numeric Evaluation; *IQR*, interquartile range; *ASES*, American Shoulder and Elbow Surgeons; *SST*, Simple Shoulder Test; *VAS*, visual analog scale.

Values are reported as mean ± standard deviation or median [interquartile range], as appropriate. Between-group comparisons were performed using independent *t*-tests or Mann–Whitney *U* tests, as appropriate.

∗ Internal rotation was scored on a 10-point scale using the following conversions: buttock/greater trochanter = 2, sacrum–L4 = 4, L1–L3 = 6, T8–T12 = 8, T1–T7 = 10.

## Discussion

The results of this study demonstrate that use of a high-speed pencil-tip burr for removal of well-fixed humeral components allows reliable stem extraction without osteotomy, with an intra-operative fracture rate of 21.1%, radiographic healing in 77.8% of fractures, and significant improvement across all patient-reported outcome measures at latest follow-up. Over a 17-year period, all well-fixed humeral stems were able to be removed without an osteotomy or cortical window using this technique. While 27 patients (21.1%) sustained an intra-operative humeral fracture, most commonly involving the greater tuberosity (15 cases), the clinical impact was minimal (Table V). At latest radiographic follow-up, 21 fractures demonstrated healing, while 4 greater tuberosity fragments displaced and 2 fragments resorbed over time.

Multiple techniques have been described for the removal of a humeral component in revision TSA. Taylor et al^12^ reported an osteotomy technique for revision TSA as a technique in which a vertical or transverse incision is made in the humerus, followed by insertion of an osteotome that is gently rotated at multiple points to open the osteotomy and facilitate separation of the humerus from the cement mantle or implant. The osteotomy is later repaired using cerclage fixation. While effective, this technique carries risks, including periprosthetic fracture, iatrogenic nerve injuries, and infection. In their series of 25 patients with an average follow-up of 65.6 months, osteotomy-related complications were observed in 2 patients: 1 sustained a periprosthetic fracture and 1 experienced cable irritation requiring cable removal at 1 year post-operatively. Van Thiel et al^14^ reported that vertical humeral osteotomy is an effective method for removing a humeral prosthesis without additional perioperative or post-operative fractures in a series of 27 patients followed for an average of 40.9 months; the technique entails using a vertical humeral osteotome at the proximal collar to interrupt the interface between the implant and the tuberosity bone to facilitate removal while preserving the tuberosities. Similarly, Johnston et al^7^ reports that a longitudinal split osteotomy caused no iatrogenic fractures and no evidence of humeral loosening or non-union in their series of 13 patients with an average follow-up of 30 months.

Sperling et al^11^ described a humeral window for revision TSA as a technique in which an anterior and/or medial cortical window is made in the humerus to allow direct access to the cement mantle or implant and facilitate disruption of the interface between the implant and the bone. Potential risks involved in this approach include intra-operative fracture, malunion, and non-union of the removed cortical piece. In their series of 20 patients with an average follow-up of 3.3 years, 4 (20%) intra-operative fractures were reported, all of which occurred during disimpaction of a partially loosened humeral component. Dodson et al^5^ report successful union and no evidence of humeral loosening or iatrogenic fracture in 7 patients using an anterior humeral window technique that employs an oscillating saw in a circumferential manner to remove a window that is one third of the circumference of the bone. Sahota reports a higher rate (23%) of intra-operative fracture, with 6 fractures (5 in the greater tuberosity and 1 in the humeral shaft) out of 26 patients that underwent creation of a humeral window, compared to 1 fracture (greater tuberosity) out of 19 patients that underwent longitudinal osteotomy.^10^

Although humeral osteotomies and cortical windows have proven to provide an effective and controlled manner for stem removal, they introduce a set of complications that may be mitigated using a high-speed burr for extraction. Creating an osteotomy involves increased soft tissue dissection and cortical violation, thus increasing the risk of non-union, fracture propagation, hematoma formation, and infection.^17^

In the present study, 27 of 128 patients (21.1%) sustained an intra-operative humeral fracture during stem extraction, despite no patient requiring an osteotomy or cortical window. This rate compares favorably with prior reports of iatrogenic fracture during humeral stem extraction even without cortical windows or osteotomies. Wall et al^16^ described a 24.1% iatrogenic fracture rate in revision procedures. Himer et al^6^ reported on 127 revision TSA with complete humeral stem removal, and found an iatrogenic humeral fracture rate of 24.4% during stem extraction, with 7.9% requiring a humerotomy. This highlights the morbidity associated with humeral component removal generally and suggests burr-assisted extraction may reduce reliance on adjunctive humerotomy compared with prior series.

The outcomes of revision TSA are generally inferior when compared to primary procedures. In a cohort of 78 revision TSAs with a mean follow-up of 43 months, Dines et al^4^ described a mean University of California, Los Angeles shoulder score of 21.4, a mean L'Insalata score of 68.7, and nearly half of cases graded as fair or poor, conveying the functional limitations observed following revisions shoulder arthroplasty. Mathew et al,^8^ in a matched cohort study, similarly reported a complication rate of 38.2% among revision TSA patients. In the present study, revision patients requiring removal of a well-fixed humeral stem demonstrated improvements in patient reported outcomes, including a mean SANE improvement of approximately 32 points. This supports the ability of burr assisted humeral stem extraction to achieve the expected functional outcomes of revision surgery, despite the technical difficulties associated with the removal of a well-fixed humeral component.

Iatrogenic fracture risk remains an important risk during removal of a well-fixed humeral stem and is seen as a complication that may compromise post-operative recovery. In the present study, all 27 patients who sustained an intra-operative humeral fracture demonstrated significant improvement in patient-reported outcomes, including ASES, SST, SANE, VAS function, and VAS pain. Forward elevation improved from a median of 55° pre-operatively to 120° post-operatively, though this did not reach significance after correction for multiple comparisons. When directly compared with patients without fracture, patient reported outcomes, forward elevation and internal rotation were similar between cohorts (Table V); Patients sustaining an intra-operative humeral fracture also demonstrated lower post-operative external rotation compared with those without fracture, though this difference did not reach statistical significance after correction for multiple comparisons (*P* = .004). Radiographically, 21 of 27 fractures (77.8%) healed, with all non–greater tuberosity fractures healing; the 6 fractures that did not heal were limited to the greater tuberosity, with 4 displaced and 2 undergoing resorption. These findings are consistent with prior reports describing favorable outcomes despite intra-operative fracture during humeral stem removal. Athwal et al^1^ reported that in 14 patients experiencing intra-operative humeral fractures during revision arthroplasty, all fractures ultimately healed; however, there was no significant improvement in range of motion despite meaningful pain relief, and fractures were associated with a high complication burden including transient nerve injuries and post-operative fracture displacement. In contrast, Wagner et al,^15^ in a retrospective analysis of 224 patients (230 shoulders) undergoing revision to rTSA, reported an intra-operative humeral fracture rate of 16%, with pain relief achieved in over 90% of cases and shoulder abduction improving from 47° to 115°; post-operative ASES and SST scores, complication rates, and implant survivorship were not significantly different between patients with and without intra-operative fracture. These results suggest that, when appropriately managed, intra-operative humeral fracture during burr-assisted removal of a well-fixed humeral stem still achieves meaningful clinical improvement, consistent with prior series.

While this study represents one of the largest series of patients treated with revision TSA in the setting of a well-fixed humeral stem, it is not without limitations. The retrospective design and single-surgeon cohort may limit the generalizability of these findings. Although all procedures were performed by a single fellowship-trained high volume shoulder arthroplasty surgeon using a consistent technique, different surgeons with varying experience, implant systems, and operative preferences may experience differing results. Without a direct comparison group, there is a lack of direct assessment of the relative effectiveness of the high-speed burr. Additionally, the high-speed burr device utilized in this study may not be universally available in all operating rooms. Outcomes were also not stratified by fixation method (cemented versus press-fit) of the revision humeral component. Despite these limitations, this study provides valuable clinical insight into the safety and efficacy of a single surgical technique for humeral stem extraction in revision TSA.

## Conclusion

The use of a high-speed pencil-tip burr for the removal of a well-fixed humeral component during revision TSA allows for reliable extraction without the need for osteotomy or cortical window, with an intra-operative fracture rate comparable to previously reported series, favorable radiographic healing of fractures when they occur, and significant improvement in clinical and patient-reported outcomes at latest follow-up.

## Disclaimers:

Funding: No funding was disclosed by the authors.

Conflicts of interest: Jonathan C. Levy is a paid consultant for Enovis, Stryker, Exactech, and Globus Medical; receives royalties from Enovis, Stryker, Exactech, Globus Medical, and Innomed; and receives research support from Enovis and Stryker. Any additional authors, their immediate families, and any research foundations with which they are affiliated have not received any financial payments or other benefits from any commercial entity related to the subject of this article.
